# Relationship between P53 Status and Response to Chemotherapy in Patients with Gastric Cancer: A Meta-Analysis

**DOI:** 10.1371/journal.pone.0095371

**Published:** 2014-04-16

**Authors:** Hai-Yuan Xu, Wen-Lin Xu, Li-Qiang Wang, Min-Bin Chen, Hui-Ling Shen

**Affiliations:** 1 Department of Medical Oncology, Kunshan First People's Hospital Affiliated to Jiangsu University, Kunshan, People's Republic of China; 2 Department of Central Laboratory, Zhenjiang Fourth People's Hospital Affiliated to Jiangsu University, Zhenjiang, People's Republic of China; 3 Department of Medical Oncology, Zhenjiang First People's Hospital Affiliated to Jiangsu University, Zhenjiang, People's Republic of China; University of Saarland Medical School, Germany

## Abstract

**Background:**

Previous studies have yielded conflicting results regarding the relationship between p53 status and response to chemotherapy in patients with gastric cancer. We therefore performed a meta-analysis to expound the relationship between p53 status and response to chemotherapy.

**Methods/Findings:**

Thirteen previously published eligible studies, including 564 cases, were identified and included in this meta-analysis. p53 positive status (high expression of p53 protein and/or a mutant p53 gene) was associated with improved response in gastric cancer patients who received chemotherapy (good response: risk ratio [RR]  = 0.704; 95% confidence intervals [CI]  = 0.550–0.903; P = 0.006). In further stratified analyses, association with a good response remained in the East Asian population (RR = 0.657; 95% CI = 0.488–0.884; P = 0.005), while in the European subgroup, patients with p53 positive status tended to have a good response to chemotherapy, although this did not reach statistical significance (RR = 0.828, 95% CI = 0.525–1.305; P = 0.417). As five studies used neoadjuvant chemotherapy (NCT) and one used neoadjuvant chemoradiotherapy (NCRT), we also analyzed these data, and found that p53 positive status was associated with a good response in gastric cancer patients who received chemotherapy-based neoadjuvant treatment (RR = 0.675, 95% CI = 0.463–0.985; P = 0.042).

**Conclusion:**

This meta-analysis indicated that p53 status may be a useful predictive biomarker for response to chemotherapy in gastric cancer. Further prospective studies with larger sample sizes and better study designs are required to confirm our findings.

## Introduction

It is estimated that gastric cancer is the fourth most common cancer in the world [1]. In 2013, an estimated 21,600 new cases will occur and 10,990 cases will eventually die of their disease in the United States [2]. Despite advances in surgical treatment and chemotherapy, prognosis remains poor, particularly as most tumors are diagnosed late and in locally advanced or advanced stages. Currently, due to the ability to shrink cancerous lesions to increase R0 resection rate, neoadjuvant chemotherapy is recommended as the standard treatment for the management of locally advanced gastric cancer [3]. Chemotherapy can also improve the outcome of unresectable gastric cancer. However, some studies suggest that only those patients who respond to neoadjuvant chemotherapy with tolerable toxicity will potentially benefit from this approach, while a proportion of patients fail to respond to neoadjuvant chemotherapy, or even progress during therapy [4]–[6]. Therefore, predictive markers to identify those patients who would benefit from neoadjuvant chemotherapy are being actively sought.

To date, p53, the most studied gene, may be the primary candidate biomarker for predicting the response of gastric cancer to chemotherapy [7]. The gene encoding p53 is located on chromosome 17p and consists of 11 exons and 10 introns. It has important cellular functions, including in cell cycle regulation, apoptosis, and DNA repair [8], [9]. p53 is the gene most frequently mutated in human cancer, with alterations occurring in at least 50% of human malignancies, playing critical roles in their development [10]. Experimental evidence suggests that p53 status is associated with tumor response to genotoxic agents [11]–[13]. However, data regarding the use of p53 status as a biological marker to predict the response of gastric cancer to chemotherapy are inconclusive [14]–[19]. Some studies found that patients with p53 mutations or overexpression had higher response rates to chemotherapy than those with normal p53 status; however, other reports drew different conclusions. Therefore, we conducted a meta-analysis to determine the value of p53 status in predicting response to chemotherapy in gastric cancer.

## Materials and Methods

### Publication Search

Studies were identified by a computerized search of the PubMed, Embase, and Web of Science databases (up to Jun 8, 2013) using the following search terms: ‘TP53’, ‘p53’, ‘p53 protein’, ‘p53 mutation’, ‘17p13 gene’, ‘chemotherapy’, ‘chemoradiotherapy’, and ‘gastric cancer’. All potentially eligible studies were retrieved and their references were carefully researched to identify other eligible studies. When multiple studies of the same patient population were identified, the published report with the largest sample size was included.

### Inclusion and Exclusion Criteria

Studies selected in this meta-analysis fulfilled all of the following criteria: (a) studies evaluating p53 status for predicting the response to chemotherapy or chemoradiotherapy in gastric cancer; (b) studies involving clinical or pathological therapeutic response; (c) retrospective or prospective cohort study; (d) studies including adequate data to allow the estimation of a risk ratio (RR) with 95% confidence intervals (95% CI); and (e) studies in English or Chinese. Reviews, letters to the editor, and articles published in books were excluded.

### Data Extraction and Definitions

Using the inclusion criteria listed above, the following information was extracted from each study: the first author's surname, the publication year, the country of origin, the number of patients analyzed, the treatment, the methods of detection, p53 positive (overexpression or mutation) rate, the type of therapeutic response, the response criteria, and the main outcomes. This information was entered in tables showing the clinical or pathological response to chemotherapy with respect to p53 status. Data was carefully extracted from all eligible publications by two investigators. Any disagreement between the investigators was resolved by discussion until a consensus was reached. If they failed to reach an agreement, a third investigator was consulted to resolve the discrepancies.

As previously reported [20], the definitions and standardizations for ‘p53’ and ‘response to therapy’ used in our study followed those of the study by Pakos et al. [21]. For consistency, we used ‘p53 status’ to refer to both gene and protein markers. p53 positive status indicates patients with high expression of p53 protein and/or mutations in the p53 gene. Good response was defined as complete response (CR) and partial response (PR), or grade 1b+2+3. Poor response was defined as stable disease (SD) and progress disease (PD), or grade 0+ 1a according to the guidelines for the clinical and pathologic studies on gastric carcinoma by the JRSGC (Japanese Research Society for Gastric Carcinoma), WHO (World Health Organization), or RECIST (Response Evaluation Criteria in Solid Tumors) criteria [22]–[25]. The response classification is detailed in Table 1.

**Table 1:** Criteria for response evaluation and standard definitions.

Criteria	Poor response	Standard definition	Complete response
		Good response	
WHO[25]	NC+PD, <50% decrease in tumor load	PR+CR, >50% decrease in tumor load	CR, disappearance of all known disease
RECIST[24]	PD+SD, <30% disease regression	PR+CR, >30% disease regression	CR, 100% disease regression
JRSGC[22], [23], [34]	PD+SD, Grade 0+1,viable cancer cells account for more than 1/3	PR, Grade 2+3, viable cancer cells account for less than 1/3	CR, Grade 3, no residual viable tumor cells
Sirak et al.[27]	Inoperable tumor after NCRT	Reduction of at least one T-stage level and/or finding of intense tumor regression on histopathologic examination	pCR, absence of tumor cells in the primary site
Cascinu et al.[28]	NR	>50% reduction in the visible tumor or complete disappearance of tumor but positive histology on biopsy of the previously involved area	Complete resolution of the endoscopically visible tumor and a negative biopsy of the original site of the tumor.
Giatromanolaki et al.[31]	25–49% reduction in tumor size	50–95% reduction in tumor size	Disappearance of a measurable lesion

WHO, World Health Organization; RECIST, Response Evaluation Criteria in Solid Tumors; JRSGC, Japanese Research Society for Gastric Carcinoma; CR, complete response; PR, partial response; PD, progressive disease; SD, stable disease; NR, no record; NC, no change.

### Statistical Analysis

The software STATA version 12 (StataCorp, College Station, TX) was used to perform the data analysis. We assessed and quantified statistical heterogeneity for each pooled estimate using the I^2^ statistic, and p>0.10 was defined as no heterogeneity. The pooled RR was calculated using a fixed-effects model (the Mantel–Haenszel method) or a random-effects model (the DerSimonian and Laird method), according to the heterogeneity results. Pooled analysis was performed using the Mantel-Haenszel model and reported as RR with 95% CIs. The significance of the pooled RR was determined by the z test and P<0.05 was considered statistically significant. χ^2^ and z represented the test statistics of the I^2^ statistic for heterogeneity and z test for the significance of the pooled RR respectively. The Begg's funnel plot and Egger's test were employed to estimate potential publication bias. We also performed sensitivity analysis by omitting each study or specific studies to find potential outliers.

## Results

### Eligible Studies

**Figure 1:**
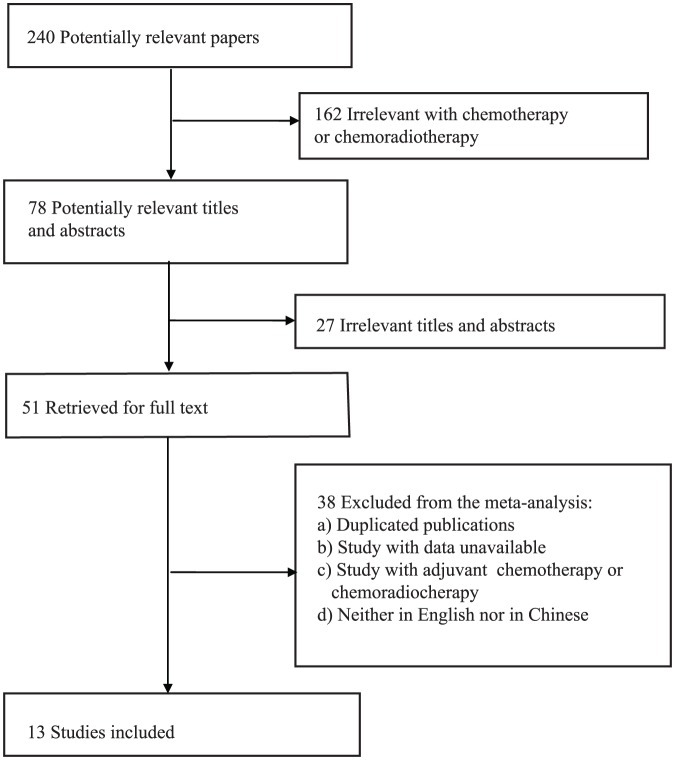
Flow diagram illustrating the screening and selection process.

Using different combinations of key terms, a total of 240 articles were retrieved by a literature search of the PubMed, Embase, and Web of Science databases. As indicated in the search flow diagram (Figure 1), 13 studies were finally included in this meta-analysis [15]–[19], [26]–[33]. The characteristics of the eligible studies are summarized in Table 2. Five used NCT, one used NCRT, and seven used CT (Table 2). The sample sizes in eligible studies ranged from 23–131 patients (median  = 36, mean  = 43, standard deviation [SD]  = 28). Overall, the eligible studies included 564 patients. Five studies were conducted in European populations (167 patients) [15], [16], [27], [28], [31], whereas eight were in East Asian populations (397 patients) [17]–[19], [26], [29], [30], [32], [33].

**Table 2:** Characteristics of studies included in the meta-analysis.

Author	Year	Country	Cases	Treatment	Detection	p53 (%)	Response	Response criteria		Standard definition response		Response rate (%)	
									Poor response	Good response	Complete response	Good response	Complete response
Qu et al.[32]	2013	China	53	NCT	IHC	53%	clinical	RECIST	PD+SD	PR	CR	53%	0
Sirak et al. [27]	2009	Czech republic	36	NCRT	IHC	63%	pathologic	Sirak et al.	Inoperable	Down-staging	pCR	47%	22%
Kamoshida et al. [26]	2007	Japan	38	NCT	IHC	39%	pathologic	JRSGC	grade 0+1a	grade 1b+2	grade 3	34%	0
Boku et al. [17]	2007	Japan	131	CT	IHC	43%	clinical	WHO/JRSGC	PD+SD	PR+CR	NR	28%	NR
Nagashima et al. [18]	2005	Japan	55	CT	IHC	44%	clinical	WHO	PD+SD	PR+CR	NR	55%	NR
Bataille et al. [16]	2003	Germany	25	NCT	IHC/gene	56%	pathologic	JRSGC	grade 0+1	grade 2	grade 3	44%	28%
Ott et al. [15]	2003	Germany	48	NCT	IHC/gene	35%	clinical	WHO	PD+SD	PR	NR	40%	NR
Giatromanolaki et al. [31]	2001	Greece	28	CT	IHC	25%	clinical	Kamoshida et al.	MR	PR	CR	36%	NR
Kikuyama et al. [30]	2001	Japan	28	CT	IHC	46%	clinical	WHO/JRSGC	PD+SD	PR	CR	36%	4%
Yeh et al. [19]	1999	Taiwan	30	CT	IHC	20%	clinical	WHO	PD+SD	PR+CR	NR	50%	NR
Boku et al. [29]	1998	Japan	39	CT	IHC	38%	clinical	WHO/JRSGC	PD+NC	PR+CR	NR	33%	NR
Cascinu et al. [28]	1998	Italy	30	NCT	IHC	53%	clinical	Cascinu et al.	NR	PR	CR	40%	10%
Nakata et al. [33]	1998	Japan	23	CT	IHC	61%	clinical	JRSGC	PD+NC	PR	CR	43%	9%

CT, chemotherapy; NCT, neoadjuvant chemotherapy; NCRT, neoadjuvant chemoradiotherapy; IHC, immunohistochemistry; WHO, World Health Organization; RECIST, Response Evaluation Criteria in Solid Tumors; JRSGC, Japanese Research Society for Gastric Carcinoma; CR, complete response; PR, partial response; PD, progressive disease; SD, stable disease; NR, no record; NC, no change.

### Relationship between p53 Status and Response to Chemotherapy in Gastric Cancer

**Figure 2:**
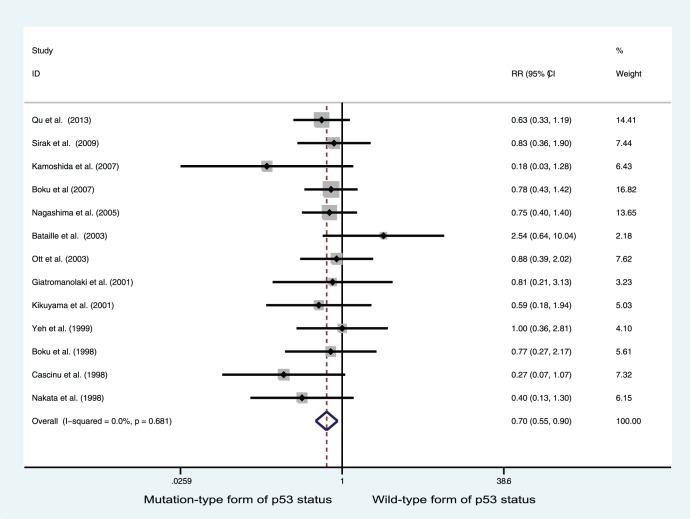
Forest plots of RR estimated for the relationship between p53 status and good response among gastric cancer patients treated with chemotherapy.

Among the studies of gastric cancer patients who received chemotherapy, 13 (involving 564 patients) contributed data to the calculation of total OR (total OR  =  clinical OR + pathological OR). p53 positive status was significantly associated with improved total OR among patients treated with chemotherapy (RR = 0.704; 95% CI = 0.550–0.903; P = 0.006, Figure 2). With respect to studies reporting both clinical and pathological responses, the latter data was used, but the clinical response data was also examined with similar results (data not shown). p53 protein expression measured by immunohistochemistry (IHC) does not directly correspond to p53 mutation detected by gene sequencing [15], [16]. As all studies included in this meta-analysis employed IHC-based protein detection, and only two employed both IHC and molecular genetic analysis, we adopted the data generated using IHC and also conducted statistical analysis for the molecular genetic data with similar results (RR = 0.720; 95% CI = 0.565–0.916; P = 0.008).

### Subgroup Analysis

**Figure 3:**
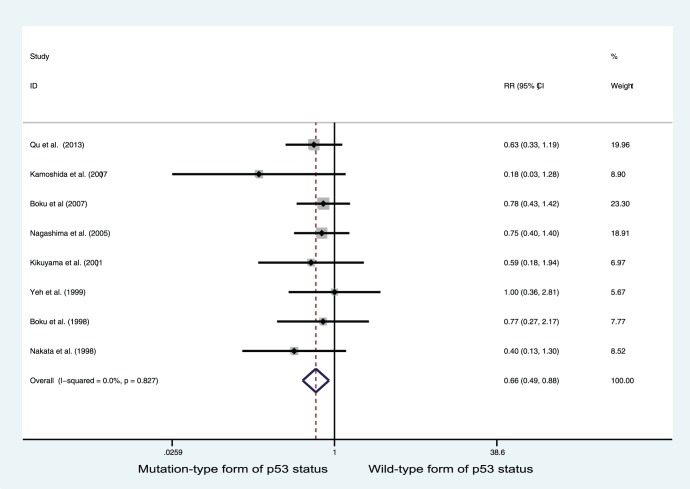
Forest plots of RR estimated for the relationship between p53 status and good response to chemotherapy in East Asian population with gastric cancer.

East Asian and European subgroups were also analyzed separately (Table 3). p53 positive status was associated with improved response in gastric cancer patients who received chemotherapy in the East Asian subgroup (RR = 0.657, 95% CI = 0.488–0.884; P = 0.005; Figure 3). In the European subgroup, however, patients with p53 positive status tended to have high response rates to chemotherapy, but the results did not reach statistical significance (RR = 0.828, 95% CI = 0.525–1.305; P = 0.417).

**Table 3:** Risk ratio for the association between p53 positive status and good response to chemotherapy.

	N	RR (95% CI)	z	P	χ^2^	Ph
All studies	13	0.704 (0.550–0.903)	2.77	0.006	9.25	0.681
**Treatment**						
CT	7	0.729 (0.525–1.013)	1.89	0.059	1.55	0.956
NCT	5	0.644 (0.422–0.985)	2.03	0.042	7.56	0.109
NCT+NCRT	6	0.675 (0.463–0.985)	2.04	0.042	7.73	0.172
**Area**						
East Asian	8	0.657 (0.488–0.884)	2.78	0.005	3.58	0.827
European	5	0.828 (0.525–1.305)	0.81	0.417	5.14	0.273
**Type of measurement**						
IHC	13	0.704 (0.550–0,903)	2.77	0.006	9.25	0.681
IHC + gene	11+2	0.720 (0.565–0.916)	2.67	0.008	9.91	0.624

Subgroup analysis was performed when at least five studies were in a subgroup.

N, number of studies; z, the test statistics of z test; P, p value of the z test; χ^2^, the test statistics of I^2^ statistic for heterogeneity; Ph, p value of the I^2^ statistic.

**Figure 4:**
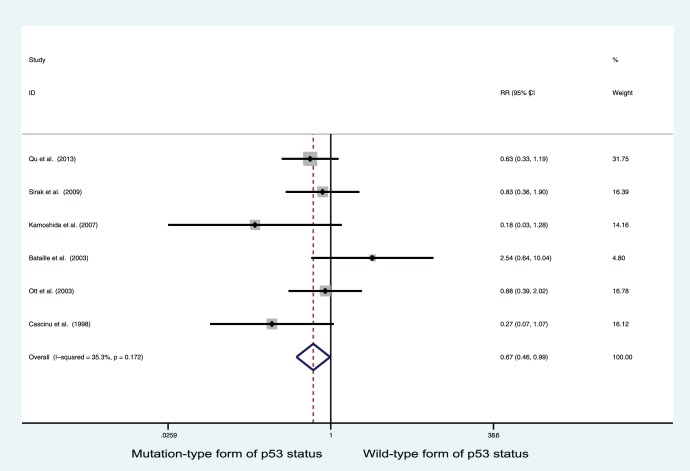
Forest plots of RR estimated for the relationship between p53 status and good response to chemotherapy-based neoadjuvant treatment in patients with gastric cancer.

As five studies used NCT and one used NCRT, we also analyzed these data, and found that p53 positive status was associated with improved response in gastric cancer patients who received chemotherapy-based neoadjuvant treatment (RR = 0.675, 95% CI = 0.463–0.985, P = 0.042; Figure 4).

### Publication Bias and Sensitivity Analysis

**Figure 5:**
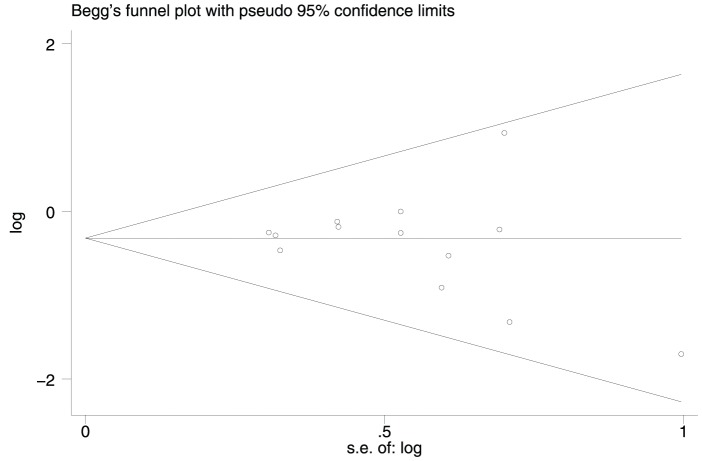
Funnel plot demonstrating that there was no obvious indication of publication bias for the outcome of good response.

The Begg's funnel plot and Egger's test were employed to estimate the publication bias of the literature included in this study. The shape of the funnel plot showed no obvious evidence of asymmetry (Figure 5), and the Egger's test indicated an absence of publication bias (P>0.05). In addition, sensitivity analysis was conducted to assess the influence of individual studies on the summary effect. No individual study dominated this meta-analysis, and the removal of any single study had no significant effect on the overall results (data not shown).

## Discussion

p53 status plays a key role in the response to many anticancer drugs. However, no consistent conclusion regarding the effect of p53 mutations on the sensitivity or resistance of gastric cancers to anticancer drugs has been reported. To date, the majority of available clinical reports involve small sample sizes, and were therefore unable to determine the value of p53 status for predicting the response to chemotherapy. Thus, we conducted a meta-analysis of 13 studies to systematically evaluate the association between p53 status and response to chemotherapy in a large population with gastric cancer.

Our results show that p53 positive status may predict response to chemotherapy in patients with gastric cancer. p53 positive status was associated with improved total OR. Stratification according to ethnicity showed that p53 positive status was significantly associated with increased OR in East Asian populations. In addition, with respect to neoadjuvant chemotherapy, our results showed that p53 positive status was associated with good response.

Although we did our utmost to perform a comprehensive analysis, some limitations remain in this study. Firstly, the meta-analysis may have been influenced by publication bias, as we limited the literature search to studies performed in English or Chinese, and we did not explore conference proceedings or abstract books. Although we attempted to identify all relevant data, some missing data are inevitable. However, using statistical methods, no publication bias was detected, suggesting that the pooled results are likely to be unbiased. Second, in this meta-analysis we used data derived from IHC-based detection of p53, which was performed in all included studies. However, the reported frequencies of positive p53 staining were variable, which may reflect the use of different antibodies, staining standards, criteria for positivity, and the inclusion of differently selected groups of gastric cancer patient groups. Third, the evaluation criterion of response to treatment among the studies was highly variable. Standardization is therefore of great importance for obtaining an accurate assessment of the clinical significance of p53 status. Despite our considerable efforts to standardize definitions, some variability among studies was inevitable. In addition, many other factors that could affect tumor sensitivity to treatment, such as tumor size, histological subtype, patient age, chemotherapy regimen, dose of chemotherapy or radiation, and courses of treatment, could not be obtained in sufficient detail for inclusion in statistical analyses. Fourth, as our analysis was observational in nature, we cannot exclude confounding as a potential explanation of the observed results.

Despite these limitations, this meta-analysis had several advantages. This is the first meta-analysis to evaluate the usefulness of p53 status for predicting the response of gastric cancer patients to chemotherapy. Also, as mentioned above, no publication bias was detected. The results showed that p53 status might be a useful predictive biomarker for evaluating response to chemotherapy in gastric cancer patients, especially in East Asian populations. However, future prospective studies with larger sample sizes, better study designs, and accurate detection methods are required to confirm our findings.

## Supporting Information

Checklist S1 PRISMA Checklist.
(PDF)
